# Detection of *Neorickettsia risticii*, the agent of Potomac horse fever, in horses from Rio de Janeiro, Brazil

**DOI:** 10.1038/s41598-020-64328-2

**Published:** 2020-04-29

**Authors:** Patrícia Gonzaga Paulino, Nádia Almosny, Renata Oliveira, Claudia da Silva, Maristela Peckle, Andresa Guimarães, Cristiane Baldani, Carlos Massard, Huarrisson Santos

**Affiliations:** 1 Department of Epidemiology and Public Health, Federal Rural University of Rio de Janeiro (UFRRJ), BR 465, Km 7, Seropedica, RJ 23890000 Brazil; 2 Department of Animal Parasitology, Federal Rural University of Rio de Janeiro (UFRRJ), BR 465, Km 7, Seropedica, RJ 23890000 Brazil; 3 Department of Veterinary Clinic and Pathology, Federal Fluminense University, Niteroi, Brazil; 4 Department of Veterinary Medicine and Surgery, Veterinary Institute, Federal Rural University of Rio de Janeiro (UFRRJ), BR 465, Km 7, Seropedica, RJ 23890000 Brazil

**Keywords:** Phylogeny, DNA sequencing, Bacterial genomics, Infectious-disease epidemiology, Infectious-disease diagnostics

## Abstract

This study aims to report the presence of *Neorickettsia risticii* DNA in blood samples from naturally infected horses in Rio de Janeiro, provide clinicopathological findings related to the infection, and report the phylogenetic diversity of the 16S rDNA of *N. risticii* in order to evaluate its heterogeneity. Real-time quantitative polymerase chain reaction (qPCR) was performed to investigate the presence of *N. risticii* in samples collected from horses (n = 187). Five positive samples were found in the molecular screening. Hypoalbuminemia and high levels of creatine kinase and lactate dehydrogenase were the predominant findings in the biochemical analysis. The sequences were similar to those of *N. risticii*. Phylogenetic analysis revealed genotype segregation based on the geographical distribution in the *N. risticii* sequence clade. Dendrograms constructed with five hypervariable regions revealed that V4 distinguished *Neorickettsia* at the species level and produced a phylogeny that best represented the phylogeny obtained with the complete 16S rDNA sequence. This is the first report of *N. risticii* DNA in the blood of Brazilian horses based on sequences deposited in GenBank. Further studies are necessary to clarify the epidemiological chain of this vector-borne parasite in order to determine and establish appropriate preventive measures in the equine trading market.

## Introduction

Potomac horse fever (PHF) is an acute systemic and potentially fatal disease of horses, which is also known as equine monocytic ehrlichiosis. It is caused by *Neorickettsia risticii*, an obligate intracellular endosymbiotic bacterium of digeneans (Platyhelminthes, Digenea) that parasitize snails and insects^1,2^. This disease is normally acquired through the accidental ingestion of insects or snails containing encysted trematodes infected with *N. risticii*^3^. Once the bacterium is released into the lumen of a horse’s gastrointestinal tract, it invades and multiplies in colon epithelial cells. It translocates into the blood and infects monocytes, mast cells, and macrophages^4^. Clinical presentation can vary from moderate to severe with non-specific clinical signs, which can include fever, lethargy, depression, laminitis, diarrhoea, and anorexia followed by severe dehydration^5^. Haematological and biochemical alterations, such as electrolyte loss, haemoconcentration, and prerenal azotemia, may also be observed in horses infected by *N. risticii*^6^.

Case descriptions from southern Brazil show that PHF may be transmitted by blood transfusion and by the ingestion of snails from the genus *Heleobia*^7^. Reports from Brazil, Uruguay, and the United States have suggested that the disease has a seasonal presentation (usually occurring in the summer and beginning of fall), although it may occur in any season depending on weather conditions^7,8^.

The disease has been geographically well reported in Canada^9^ and the United States^1,6^. Additionally, the DNA of *N. risticii* has been detected in bats from Argentina in South America^10,11^. In Brazil, the pathogen has been detected only at the molecular level in horses from the South Region^2–13^. Although the circulation of this pathogen has been confirmed in the South Region of Brazil, no *N. risticii* sequences are available in GenBank, and the presence of *N. risticii* DNA in other Brazilian regions has not been confirmed. Some serological studies have suggested that *N. risticii* circulates in horses in the states of Sao Paulo and Rio de Janeiro (Southeast Region)^14–16^. However, due to the possibility of cross-reaction with other organisms from the Anaplasmataceae family, the confirmation of findings using DNA detection-based methods is required.

The 16S rDNA is key to the taxonomic organization of the Anaplasmataceae family. The *Neorickettsia* group is the most divergent genetic cluster of the Ehrlichieae^17^. For example, in *N. risticii* sequences, 16S rDNA may be divergent by as many as 15 nucleotides, highlighting the phylogenetic heterogeneity of this group^17^. The 16S rDNA is highly conserved and usually has more than 97% sequence identity^18^. This 3% divergence is concentrated mainly in nine hypervariable regions that are taxon-specific and need to be determined for novel organisms by sequence analysis of the complete molecule^18^. However, only a few studies have employed complete 16S rDNA sequences due to the quality restraint of sequencing technology. Hence, the most suitable hypervariable region of 16S rDNA is important for phylogenetic studies of bacteria.

In areas where *N. risticii* circulates, it has a great economic impact on equine production. Local veterinarians need to know that PHF occurs so that they may consider it as a differential diagnosis for fever, depression, diarrhoea, and anorexia^19^. Therefore, it is very important to investigate, report, and confirm cases in areas where the disease status is unknown in order to apply preventive measures. Hence, the present study aims to detect *N. risticii* at the molecular level in whole blood samples of naturally infected horses from the state of Rio de Janeiro, Brazil, provide clinicopathological findings related to the infection, and report the phylogenetic diversity of the 16S rDNA of *N. risticii* in order to evaluate its heterogeneity.

## Results

**Figure 1:**
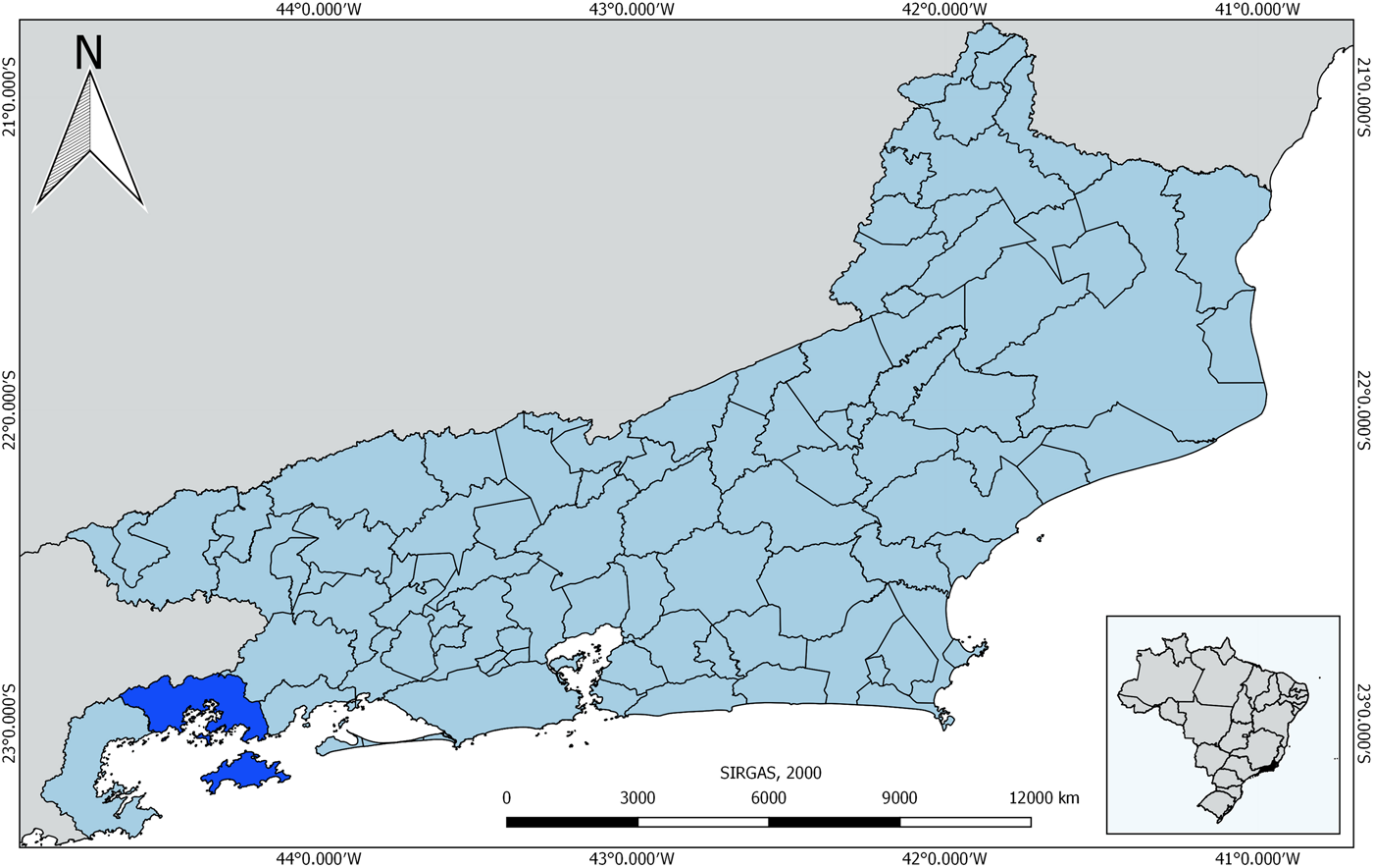
The state of Rio de Janeiro, Brazil and the location of the Angra dos Reis municipality, where blood samples were collected from horses. The colour Blue indicates the municipality of Angra dos Reis and in Grey are the bordering states. Scale bar = 20 Km.

The frequency of *N. risticii* in horses from Angra dos Reis in the state of Rio de Janeiro (Fig. 1) in the Southeast Region of Brazil was 2.67% (5/188). All positive samples were successfully sequenced and presented 99.53% to 100% similarity to other *N. risticii* sequences from GenBank and the SILVA database. The percent identity between the sequences obtained in the present study ranged from 99.92 to 100%.

Regarding clinicopathological findings, infected animals did not show abnormalities in the erythrogram, except that one presented with anaemia and thrombocytopenia according to the reference values (Supplementary Table S1). However, 60% (3/5) of infected horses showed high levels of fibrinogen. Among these horses, one presented with leukocytosis, eosinophilia, and basophilia. Another presented with lymphocytosis, and the final animal presented with only eosinophilia (Supplementary Table S1). Biochemical analysis showed that all infected horses presented with hypoalbuminemia and high Lactate Dehydrogenase and Creatine Kinase activity, while two showed hypoproteinemia (Supplementary Table S1). Only one horse had hyperglobulinemia. Urea, creatinine, and aspartate aminotransferase (AST) were within the reference range in all positive animals.

**Figure 2:**
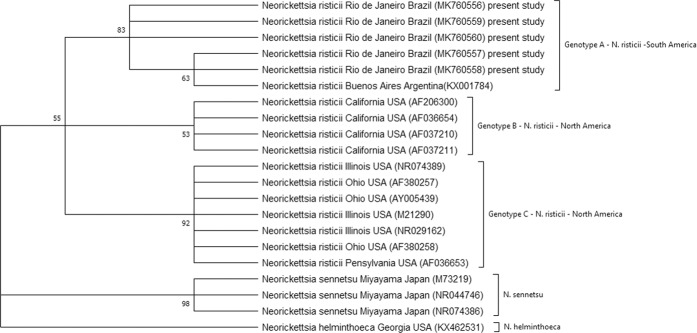
Phylogenetic analysis of the *Neorickettsia risticii* based on 16S rDNA sequence comparison. GenBank accession numbers have been shown in parentheses. The tree was constructed using the maximum likelihood method, and numbers above internal nodes indicate the percentages of 1000 bootstrap replicates that supported the branch.

The phylogenetic analysis of *N. risticii* based on 16S rDNA clearly showed that the five sequences found in this study were grouped with other *N. risticii* sequences deposited in GenBank. Based on an evolutionary distance of 0.3%, the *N. risticii* group showed three geographically distinguishable genotypes. These were Genotype A (six sequences), Genotype B (four sequences), and Genotype C (seven sequences; Fig. 2). Genotype A has composed of *N. risticii* sequences from Brazil and Argentina; Genotype B was composed of *N. risticii* sequences from Illinois, Ohio, and Pennsylvania in the United States; and Genotype C was composed of *N. risticii* from California in the United States. The *N. risticii* genotype identified in South America (that is, the one identified in Brazil and Argentina) was more closely related to the California genotype (0.3% distance) than to the Illinois, Ohio, and Pennsylvania genotype (0.5% distance) (Supplementary Fig. S1).

**Figure 3:**
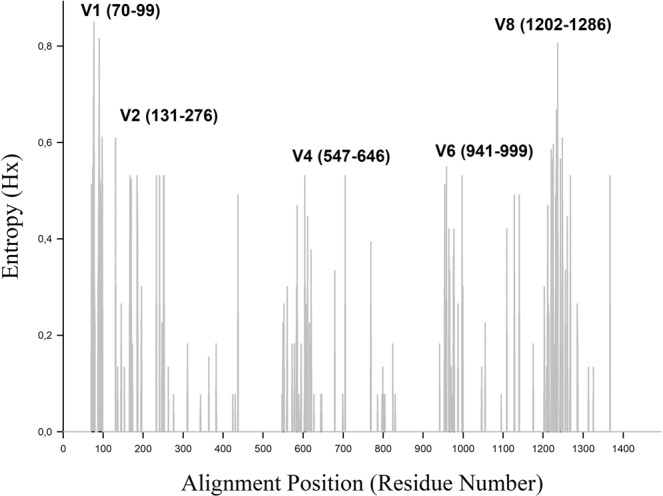
Entropy plot of 16S rRNA gene sequence alignment from *Neorickettsia risticii*. Hypervariable regions have been indicated per Chakravorty *et al*. (2007). The sequence area presented excluded poorly supported areas from the beginning and end of the sequences and thus excluded the V9 region.

Entropy analysis revealed five hypervariable regions (V1, V2, V4, V6, and V8) in the 16S rDNA sequence alignment of *Neorickettsia* spp. (Fig. 3). Dendrograms constructed with the five hypervariable regions revealed that V4 was the region that distinguished *Neorickettsia* at the species level and produced the phylogeny that best represented that obtained from the complete 16S rDNA sequence (Supplementary Figs. S2–S6). Hypervariable regions V1, V2, V6, and V8 were less suitable for species identification due to a higher degree of sequence conservation. No region could distinguish the *N. risticii* genotypes. It was impossible to determine the variable region V9 of *Neorickettsia* due to a lack of nucleotide sequences in the 5′ terminal portions.

## Discussion

The present study is the first to report the presence of *N. risticii* DNA in naturally infected horses in the Southeast Region of Brazil, in the state of Rio de Janeiro. Previous studies performed in the South Region of Brazil reported the presence of *N. risticii* in horses^8–13^. However, these reports were based on serology or PCR methods without further confirmation from either sequencing or culturing of the bacterium^2^. Although serological studies have been conducted in several localities in the Southeast Region of Brazil, to date, there have been no molecular reports confirming the presence of *N. risticii* DNA in animal samples. According to the literature, 22.2% of seroreactive horses were found in the mountains and metropolitan areas of Rio de Janeiro, and 26% of seroreactive horses were found in the Itaguai microregion^14–16^. The frequency of *N. risticii* reported in southern Brazil through molecular tests such as nested PCR was 1.75%^13^, a frequency close to that observed in the present study (2.67%, n = 5/187).

The *N. risticii-*positive horses found in the state of Rio de Janeiro presented some clinicopathological abnormalities that were compatible with PHF signs described in the literature. The pathological process of *N. risticii* causes lesions in the intestinal musculature, which may explain the biochemical abnormalities in the horses’ blood, such as the high levels of CK and high activity of LDH in all infected animals. These injuries may also cause leakage of albumin through the inflamed gut wall into the lumen, which may explain the hypoalbuminemia in all infected horses. Hypoalbuminemia and high levels of CK were previously observed in animals with PHF^6^. In that report, the significance of CK activity in the pathology was unclear, but decreased tissue perfusion may lead to acute muscle injury. Fibrinogen increases in the acute phase response and may occur as a result of inflammation of the lesions caused by the pathological process of *N. risticii*, which also leads to leukocytosis in some cases^9^. Although the biochemical changes in positive animals are marked, there is no significant difference when compared to the negative population. In this context, is possible that *N. risticii* infection in horses from the Southeast region is asymptomatic or presents minor clinical or/and laboratory changes.

The phylogenetic analysis showed that the sequences obtained from horses in Rio de Janeiro clustered together with other *N. risticii* sequences previously reported in bats (*Tadarida brasiliensis*) from Buenos Aires, Argentina^12^. These data suggested that the genotype of the bacterium does not seem to be influenced by the host. The evolutionary model produced showed that the *N. risticii* clade was composed of three distinct subclades: Genotypes A, B, and C. The genetic divergence of *N. risticii* sequences seemed to be related to the geographical location of the samples since a new third genotype from South America, which diverged from the 2 genotypes from North America, was detected in the present study. Previous studies reported such geographical segregation in the United States, showing that sequences from California and Oregon were distinct from sequences from Ohio, Maryland, and Pennsylvania and the sequence from Bueno Aires formed its own cluster^12,20,21^. Thus, genotype seems to be influenced by the unique environmental conditions of each area.

The 16S rDNA is widely used for bacterial disease diagnosis^18^. Additionally, the 16S rDNA sequence is highly conserved, which allows the creation of a bacterial profile that increases the understanding of the relationship between the genetic divergence and geographic distribution of *N. risticii*. For this reason, the current study chose to characterize the *N. risticii* samples from Brazil with this molecular marker. However, further studies targeting other molecular markers, such as 51 kDa protein (p51), the *groESL* operon and the *citrate synthase* gene (*gltA*), are necessary to improve the genetic characterization of Brazilian samples^11^.

Complete 16S rDNA gene sequences present nine hypervariable regions that are separated by conserved regions^22^. For the hypervariable regions of the 16S rDNA sequence to be useful for taxonomic classification and/or the identification of an organism at the species level, two divergent organisms cannot have identical or highly similar regions. In microbial community studies, the V3 and V6 regions are important in hypervariable regions for the taxonomic identification and distinction of bacterial groups at the genus level^23^. In the present study, the hypervariable region V4 distinguished the different *Neorickettsia* species, as evidenced by the dendrograms constructed according to the hypervariable region (Supplementary Figs. S2–S6). However, this region was unable to distinguish *N. risticii* at the genotype level, as evidenced by the phylogenetic reconstruction using the full-length sequences (Fig. 2). In this case, V4 performed well in distinguishing *Neorickettsia* at the species level. The inability of the V4 region to distinguish *N. risticii* at the genotype level may have been related to the substantial number of point polymorphisms outside of variable regions that, by default, lead to a loss of information^24^. The method of using geodesic distance to analyse the V4 hypervariable region of 16S rDNA sequences was the most reliable for representing complete sequences in phylogenetic analysis in previous studies, which was also observed in this study using the 16S rDNA sequences of *Neorickettsia* spp.^25^.

Available data regarding Potomac horse fever are scarce in Brazil, probably because aetiologies for equine enterocolitis has been poorly investigated. Confirmation of the presence of *N. risticii* in horses from the state of Rio de Janeiro is important for raising awareness of the agent, which is in circulation in the Southeast Region of Brazil and highlights the importance of implementing adequate preventive measures to reduce economic losses. Besides, this report shows the need for additional studies to gather further knowledge in order to elucidate the ecology, transmission, and epidemiology of *N. risticii*, as there is no concise information on the biological cycle involved in the transmission of this pathogen in the region.

## Material and Methods

### Animals and sampling procedures

A total of 187 blood samples were collected from horses that participated in the herbivorous rabies vaccination campaign carried out by the Angra dos Reis municipal government (−23°00′24.01″S; −44°19′5.02″W) in Rio de Janeiro in July and August of 2012 (Fig. 1). After receiving the consent of the animal’s owner, one tube containing ethylenediaminetetraacetic acid (EDTA) was used to collect a blood sample from each animal. Then, pathogen screening using molecular methods was performed. A tube without EDTA was used to obtain serum samples.

### Haematological analysis

Haematological analysis was performed using a Poch-100iv haematology analyzer (Roche, United States) to obtain horses’ red blood cell count (RBC), haemoglobin (Hb), haematocrit (HT), mean corpuscular volume (MCV), mean corpuscular haemoglobin concentration (MCHC), number of platelets (PLT), and white blood cell count (WBC). Blood smears were stained using Diff-Quick stain (Hemacolor, Merck, Brazil) and examined under a light microscope for specific leucometry. The determination of fibrinogen (FIBRI) was performed through manual refractometry using precipitation by heating samples in a water bath at 57 °C for three minutes^26^.

### Biochemical analysis

Samples collected without EDTA were centrifuged at 3000 rpm for 10 min to obtain the serum. Biochemical measurements were taken using a semi-automatic spectrophotometer (Bioplus 2000®, Barueri, SP, Brazil) using commercial kits (Labtest, Lagoa Santa, MG, Brazil). The following parameters were analysed: aspartate aminotransferase (AST), creatine kinase (CK), lactate dehydrogenase (LDH), total protein (TP), albumin, urea, and creatinine. For internal quality control of biochemical dosages in the Bioplus 2000®, the control serum Qualitrol 1 H (Labtest) was used.

### DNA extraction

DNA was extracted from EDTA blood samples using a GFX Genomic Blood Purification Mini Spin Mini Kit (GE Healthcare Life Science, SP, Brazil) following the manufacturer’s recommendations. Quantification was performed through spectrophotometry using a NanoDrop® 2000C instrument (Thermo Scientific, SP, Brazil).

### Standard controls

The *N. risticii*-positive standard control was acquired from commercial slides prepared for immunofluorescence (Fuller Laboratories, Fullerton, CA, USA). Genomic material on the slides was purified using a DNeasy Blood and Tissue Kit (Qiagen, Valencia, CA, USA) according to the manufacturer’s recommendations.

Nuclease-free water (Ambion®, Thermo Scientific, Wilmington, DE, USA) and a negative equine blood sample were used as a negative amplification control.

### Molecular detection

To perform initial pathogen screening, DNA samples extracted from equine whole blood were submitted to *N. risticii*-specific real-time quantitative PCR (qPCR) with a detection limit of 10 copies of *N. risticii* 16S rDNA^27^. The oligonucleotide probes used were the forward probe ER.133f (5′-GTTATTCCCTACTACCAGGCAAGTTC-3′), the reverse probe ER.54r (5′-AACGGAATCAGGGCTGCTT-3′), and the quencher-labelled probe ER.77p (FAM-ACGCACCCGTCTGCCACGGGA-TAMRA). All samples were tested using a *TaqMan*® Exogenous Internal Positive Control Reagents Kit (Applied Biosystems, Foster City, CA) following the manufacturer’s instructions. Reactions were performed using the following reagents in a total volume of 12 μl: 1X *TaqMan* Universal PCR Master Mix (Applied Biosystems), 400 nM each primer, 800 nM probe, and 1 μl of target DNA. The amplification conditions were as follows: 5 min at 95 °C and 45 cycles of 15 s at 95 °C and 60 s at 60 °C in a Step One instrument (Applied Biosystems). Samples with cycle quantification (“Cq”) less than or equal to 40 were considered positive.

The five positive samples from qPCR were submitted to conventional PCR to amplify the complete 16S rDNA region (1470 bp)^28^. The oligonucleotide probes used were the forward probe N16-25F (5′-TCAGAACGAACGCTAGCGGT-3′) and the reverse probe N1500R (5′-AAAGGAGGTAATCCAGCCGCAGGTTCAC-3′). The amplification conditions were as follows: 5 min at 95 °C, followed by 40 cycles of 15 s at 95 °C, 60 s at 58 °C, and 45 s at 72 °C, on a Veriti instrument (Applied Biosystems).

### Sequencing

Amplicons were purified with a Clean Sweep enzymatic purification kit (Applied Biosystem) according to the manufacturer’s recommendations. Sequencing was conducted using the Sanger method with an ABI 3730 DNA analyzer (Applied Biosystems).

### Phylogenetic analysis

The five sequences obtained were analysed with DNA Sequence Assembler v4 (Heracle BioSoft, Arges, Romania) and submitted to the Basic Local Alignment Search Tool (BLAST) algorithm to verify the similarity of the samples to nucleotide sequences available in GenBank. An initial dataset was assembled with the partial sequences of 16S rDNA obtained in this study and a set of nearly complete sequences of *Neorickettsia* spp. (1455-1342 bp). Sequences were aligned using the *ClustalW* algorithm in MEGA7 software (Molecular Evolutionary Genetics Analysis Version 7.0 for Bigger Datasets)^29^. The complete 16S rDNA sequence of *N. risticii* accession number NR074389 was used as a reference for alignment.

Phylogenetic analysis was performed using the maximum likelihood method based on the Kimura 2-parameter model^30^. The tree with the largest log-likelihood (−2088.28) is shown in Fig. 2. There were a total of 1282 positions in the final dataset. A bootstrap test with 1000 pseudo-replicates was used for the confidence analysis of the clades. The 16S rDNA sequence of *Neorickettsia helminthoeca* (KX462531) was used as an outgroup for the phylogenetic analysis. In this study, monophyletic clades with 0.3% evolutionary divergence between clades and large bootstrap values were considered distinct genotypes of *N. risticii*. Evolutionary analyses were conducted in MEGA X.

A second dataset was assembled with only 16S rDNA sequences from *Neorickettsia* spp. of over 1200 bp available in GenBank and the SILVA database. Phylogenetic reconstruction was performed using the same methods described above. In this dataset, variable regions of 16S rDNA fragments of *Neorickettsia* were studied. The 16S rDNA regions were determined according to a previously published protocol, followed by a visual analysis of the sequence alignment of *Neorickettsia* species^25^. Hypervariable regions were identified through entropy analysis using BioEdit version 7.0.9.0^31^. After alignment, a dendrogram was built using the maximum likelihood method based on the Kimura 2-parameter model for each variable region. The hypervariable region of the 16S rDNA sequence of *Neorickettsia* spp. that best represented the phylogenetic reconstruction obtained with the complete 16S rDNA sequence was also determined.

### Ethical statement

All experimental protocols used in this study were approved by the Committee on Ethics in the Use of Animals (CEUA) of the Federal University of Fluminense under number 66/11. In addition, all methods applied in this investigation were in accordance with relevant guidelines and regulations of the Rural Federal University of Rio de Janeiro.

## Supplementary information

Supplementary Information.

Dataset 1.

## Data Availability

Sequences were deposited in GenBank with accession numbers from MK760556 to MK760560. https://www.ncbi.nlm.nih.gov/nuccore/MK760556.1/; https://www.ncbi.nlm.nih.gov/nuccore/MK760557.1/; https://www.ncbi.nlm.nih.gov/nuccore/MK760558.1/; https://www.ncbi.nlm.nih.gov/nuccore/MK760559.1/; https://www.ncbi.nlm.nih.gov/nuccore/MK760560.1/.
