# Author Correction: Detection of *Neorickettsia risticii*, the agent of Potomac horse fever, in horses from Rio de Janeiro, Brazil

**DOI:** 10.1038/s41598-020-69584-w

**Published:** 2020-07-29

**Authors:** Patrícia Gonzaga Paulino, Nádia Almosny, Renata Oliveira, Vanessa Viscardi, Ananda Müller, Andresa Guimarães, Cristiane Baldani, Claudia da Silva, Maristela Peckle, Carlos Massard, Huarrisson Santos

**Affiliations:** 1 Department of Epidemiology and Public Health, Federal Rural University of Rio de Janeiro (UFRRJ), BR 465, Km 7, Seropedica, RJ 23890000 Brazil; 2 Department of Veterinary Clinic and Pathology, Federal Fluminense University, Niteroi, Brazil; 3 Ross University School of Veterinary Medicine, Basseterre, Saint Kitts and Nevis; 4 Instituto de Ciencias Clínicas Veterinarias, Universidad Austral de Chile, Valdivia, Chile; 5 Department of Veterinary Medicine and Surgery, Veterinary Institute, Federal Rural University of Rio de Janeiro (UFRRJ), BR 465, Km 7, Seropedica, RJ 23890000 Brazil; 6 Department of Animal Parasitology, Federal Rural University of Rio de Janeiro (UFRRJ), BR 465, Km 7, Seropedica, RJ 23890000 Brazil

Correction to: *Scientific Reports* https://doi.org/10.1038/s41598-020-64328-2, published online 29 April 2020

Vanessa Viscardi and Ananda Müller were omitted from the author list in the original version of this Article. This has been corrected in the PDF and HTML versions of the Article, and in the accompanying Supplementary Information files.

As a result, the Affiliations list is now as follows:

Affiliation 1:

Department of Epidemiology and Public Health, Federal Rural University of Rio de Janeiro (UFRRJ), BR 465, Km 7, Seropedica, RJ 23890000, Brazil

Patrícia Gonzaga Paulino and Huarrisson Santos

Affiliation 2:

Department of Veterinary Clinic and Pathology, Federal Fluminense University, Niteroi, Brazil

Nádia Almosny, Renata Oliveira and Vanessa Viscardi

Affiliation 3:

Ross University School of Veterinary Medicine, Basseterre, Saint Kitts and Nevis

Ananda Müller

Affiliation 4:

Instituto de Ciencias Clínicas Veterinarias, Universidad Austral de Chile, Valdivia, Chile

Ananda Müller

Affiliation 5:

Department of Veterinary Medicine and Surgery, Veterinary Institute, Federal Rural University of Rio de Janeiro (UFRRJ), BR 465, Km 7, Seropedica, RJ 23890000, Brazil

Andresa Guimarães and Cristiane Baldani

Affiliation 6:

Department of Animal Parasitology, Federal Rural University of Rio de Janeiro (UFRRJ), BR 465, Km 7, Seropedica, RJ 23,890,000, Brazil

Claudia da Silva, Maristela Peckle and Carlos Massard

Additionally, the Author Contributions section now reads:

C.M., N.A., AM originally formulated the idea, R.O., A.M. and V.V. collected samples, R.O. and A.M. performed the Complete blood count (CBC) and biochemistry measurements, C.S. and M.P. conducted extractions and sequencing, C.B. and A.G. analysed the data and P.G.P. and H.S. wrote the manuscript.

