# Supplementary material for: Detection of Neorickettsia risticii, the agent of Potomac horse fever, in horses from Rio de Janeiro, Brazil
Source: Sci Rep. 2020 Apr 29;10:7208. doi: 10.1038/s41598-020-64328-2 (PMC7190851; doi:10.1038/s41598-020-64328-2)
Supplement: Supplementary file 1 — Supplementary Information. [file 41598_2020_64328_MOESM1_ESM.pdf]

Scientific Reports

Detection of *Neorickettsia risticii*, the agent of Potomac horse fever, in horses from Rio de Janeiro, Brazil

Patrícia Gonzaga Paulino[a,*], Nádia Almosny[c], Renata Oliveira[c], Claudia da Silva[b], Maristela Peckle[b], Andresa Guimarães[d], Cristiane Baldani[d], Carlos Massard[b], Huarrisson Santos[a]

[a] Department of Epidemiology and Public Health, Federal Rural University of Rio de Janeiro (UFRRJ), BR 465, Km 7, Seropedica, RJ 23890000, Brazil.

[b] Department of Animal Parasitology, Federal Rural University of Rio de Janeiro (UFRRJ), BR 465, Km 7, Seropedica, RJ 23890000, Brazil.

[c] Department of Veterinary Clinic and Pathology, Federal Fluminense University, Niteroi, Brazil.

[d] Department of Veterinary Medicine and Surgery, Veterinary Institute, Federal Rural University of Rio de Janeiro (UFRRJ), BR 465, Km 7, Seropedica, RJ 23890000, Brazil.

[*]**Corresponding Author:** Patrícia Gonzaga Paulino, Department of Epidemiology and Public Health, Veterinary Institute, Federal Rural University of Rio de Janeiro, BR 465, Km 7, Rio de Janeiro, Seropedica 23890-000, Brazil. patgpaulino@gmail.com

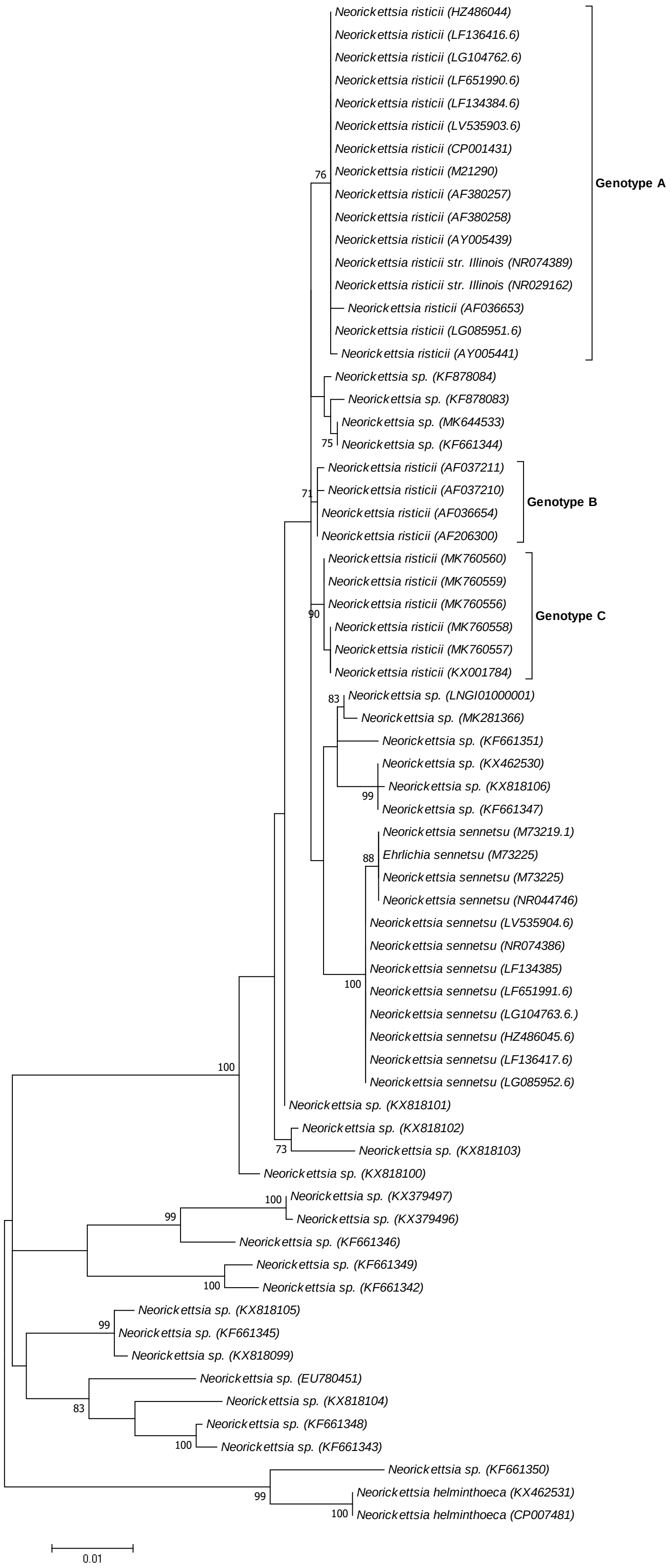
Neorickettsia risticii (HZ486044)
Neorickettsia risticii (LF136416.6)
Neorickettsia risticii (LG104762.6)
Neorickettsia risticii (LF651990.6)
Neorickettsia risticii (LF134384.6)
Neorickettsia risticii (LV535903.6)
Neorickettsia risticii (CP001431)
Neorickettsia risticii (M21290)
Neorickettsia risticii (AF380257)
Neorickettsia risticii (AF380258)
Neorickettsia risticii (AY005439)
Neorickettsia risticii str. Illinois (NR074389)
Neorickettsia risticii str. Illinois (NR029162)
Neorickettsia risticii (AF036653)
Neorickettsia risticii (LG085951.6)
Neorickettsia risticii (AY005441)
76
Genotype A
Neorickettsia sp. (KF878084)
Neorickettsia sp. (KF878083)
Neorickettsia sp. (MK644533)
75
Neorickettsia sp. (KF661344)
Neorickettsia risticii (AF037211)
71
Neorickettsia risticii (AF037210)
Neorickettsia risticii (AF036654)
Neorickettsia risticii (AF206300)
Genotype B
Neorickettsia risticii (MK760560)
Neorickettsia risticii (MK760559)
Neorickettsia risticii (MK760556)
90
Neorickettsia risticii (MK760558)
Neorickettsia risticii (MK760557)
Neorickettsia risticii (KX001784)
Genotype C
83
Neorickettsia sp. (LNGI01000001)
Neorickettsia sp. (MK281366)
Neorickettsia sp. (KF661351)
Neorickettsia sp. (KX462530)
Neorickettsia sp. (KX818106)
99
Neorickettsia sp. (KF661347)
Neorickettsia sennetsu (M73219.1)
Ehrlichia sennetsu (M73225)
88
Neorickettsia sennetsu (M73225)
Neorickettsia sennetsu (NR044746)
Neorickettsia sennetsu (LV535904.6)
Neorickettsia sennetsu (NR074386)
Neorickettsia sennetsu (LF134385)
100
Neorickettsia sennetsu (LF651991.6)
Neorickettsia sennetsu (LG104763.6.)
Neorickettsia sennetsu (HZ486045.6)
Neorickettsia sennetsu (LF136417.6)
Neorickettsia sennetsu (LG085952.6)
100
Neorickettsia sp. (KX818101)
Neorickettsia sp. (KX818102)
73
Neorickettsia sp. (KX818103)
Neorickettsia sp. (KX818100)
100
Neorickettsia sp. (KX379497)
99
Neorickettsia sp. (KX379496)
Neorickettsia sp. (KF661346)
Neorickettsia sp. (KF661349)
100
Neorickettsia sp. (KF661342)
Neorickettsia sp. (KX818105)
99
Neorickettsia sp. (KF661345)
Neorickettsia sp. (KX818099)
Neorickettsia sp. (EU780451)
83
Neorickettsia sp. (KX818104)
Neorickettsia sp. (KF661348)
100
Neorickettsia sp. (KF661343)
Neorickettsia sp. (KF661350)
99
Neorickettsia helminthoeca (KX462531)
100
Neorickettsia helminthoeca (CP007481)
0.01

Supplementary Fig. S1. Phylogenetic analysis of *Neorickettsia* spp. isolated from horses based on 16S rDNA sequence. GenBank accession numbers have been shown in parentheses. The tree was constructed using the maximum likelihood method, and numbers above the internal nodes indicated the percentages of 1000 bootstrap replicates that supported the branch.

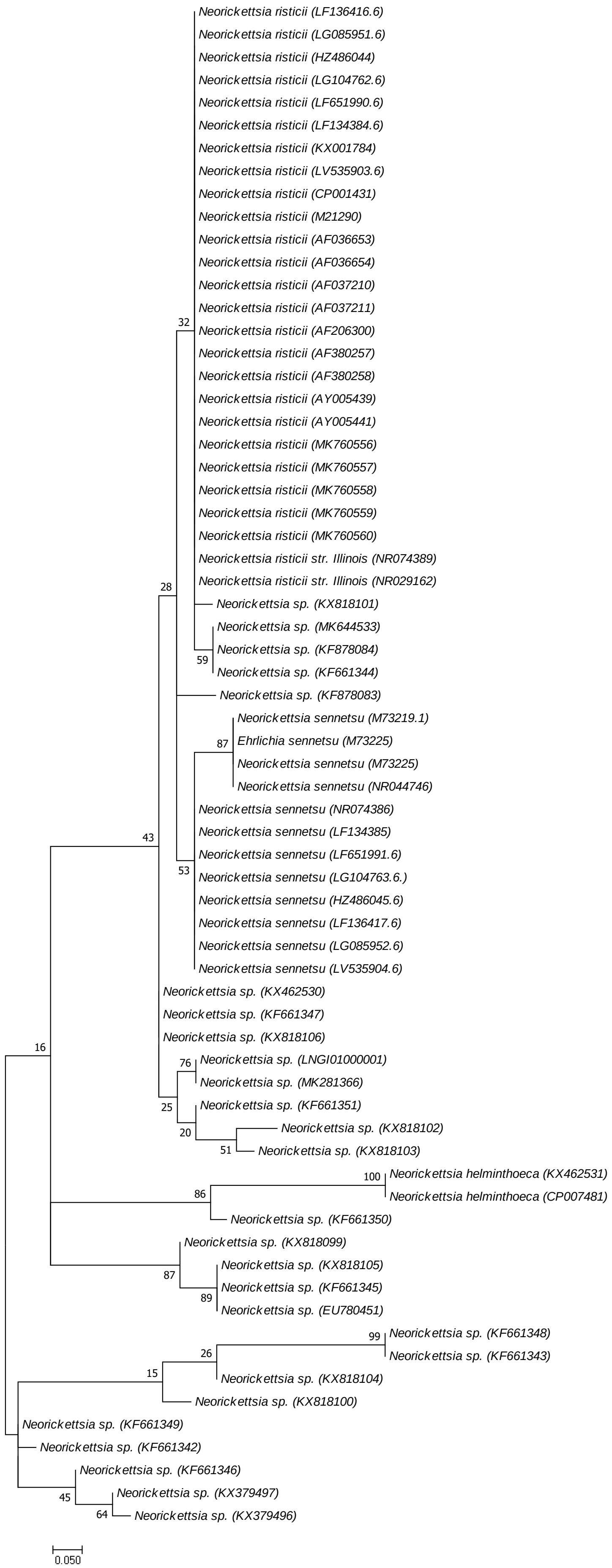
Neorickettsia risticii (LF136416.6)
Neorickettsia risticii (LG085951.6)
Neorickettsia risticii (HZ486044)
Neorickettsia risticii (LG104762.6)
Neorickettsia risticii (LF651990.6)
Neorickettsia risticii (LF134384.6)
Neorickettsia risticii (KX001784)
Neorickettsia risticii (LV535903.6)
Neorickettsia risticii (CP001431)
Neorickettsia risticii (M21290)
Neorickettsia risticii (AF036653)
Neorickettsia risticii (AF036654)
Neorickettsia risticii (AF037210)
Neorickettsia risticii (AF037211)
Neorickettsia risticii (AF206300)
Neorickettsia risticii (AF380257)
Neorickettsia risticii (AF380258)
Neorickettsia risticii (AY005439)
Neorickettsia risticii (AY005441)
Neorickettsia risticii (MK760556)
Neorickettsia risticii (MK760557)
Neorickettsia risticii (MK760558)
Neorickettsia risticii (MK760559)
Neorickettsia risticii (MK760560)
Neorickettsia risticii str. Illinois (NR074389)
Neorickettsia risticii str. Illinois (NR029162)
Neorickettsia sp. (KX818101)
Neorickettsia sp. (MK644533)
Neorickettsia sp. (KF878084)
Neorickettsia sp. (KF661344)
Neorickettsia sp. (KF878083)
Neorickettsia sennetsu (M73219.1)
Ehrlichia sennetsu (M73225)
Neorickettsia sennetsu (M73225)
Neorickettsia sennetsu (NR044746)
Neorickettsia sennetsu (NR074386)
Neorickettsia sennetsu (LF134385)
Neorickettsia sennetsu (LF651991.6)
Neorickettsia sennetsu (LG104763.6.)
Neorickettsia sennetsu (HZ486045.6)
Neorickettsia sennetsu (LF136417.6)
Neorickettsia sennetsu (LG085952.6)
Neorickettsia sennetsu (LV535904.6)
Neorickettsia sp. (KX462530)
Neorickettsia sp. (KF661347)
Neorickettsia sp. (KX818106)
Neorickettsia sp. (LNGI01000001)
Neorickettsia sp. (MK281366)
Neorickettsia sp. (KF661351)
Neorickettsia sp. (KX818102)
Neorickettsia sp. (KX818103)
Neorickettsia helminthoeca (KX462531)
Neorickettsia helminthoeca (CP007481)
Neorickettsia sp. (KF661350)
Neorickettsia sp. (KX818099)
Neorickettsia sp. (KX818105)
Neorickettsia sp. (KF661345)
Neorickettsia sp. (EU780451)
Neorickettsia sp. (KF661348)
Neorickettsia sp. (KF661343)
Neorickettsia sp. (KX818104)
Neorickettsia sp. (KX818100)
Neorickettsia sp. (KF661349)
Neorickettsia sp. (KF661342)
Neorickettsia sp. (KF661346)
Neorickettsia sp. (KX379497)
Neorickettsia sp. (KX379496)
32
28
59
87
43
53
16
76
25
20
51
100
86
87
89
99
26
15
45
64
0.050

Supplementary Fig. S2. Phylogenetic analysis of *Neorickettsia* spp. isolated from horses based on the V1 hypervariable region of the 16S rDNA sequence. GenBank accession numbers have been shown in parentheses. The tree was constructed using the maximum likelihood method, and numbers above the internal nodes indicated the percentages of 1000 bootstrap replicates that supported the branch.

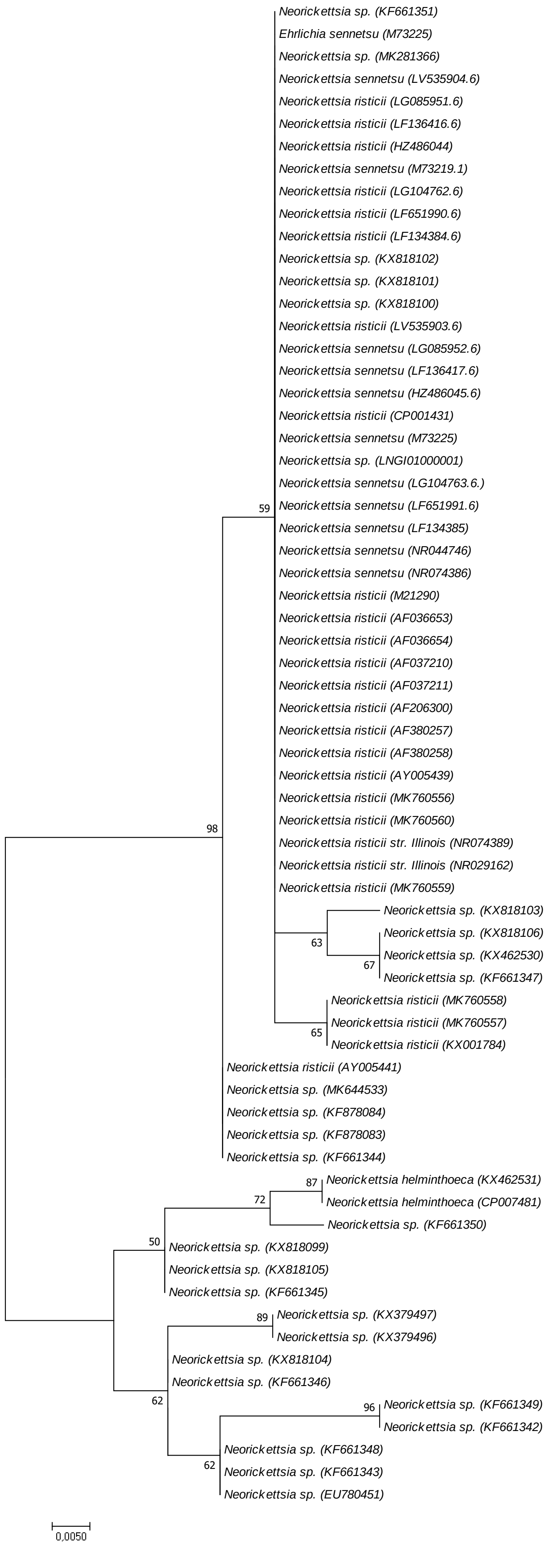
Neorickettsia sp. (KF661351)
Ehrlichia sennetsu (M73225)
Neorickettsia sp. (MK281366)
Neorickettsia sennetsu (LV535904.6)
Neorickettsia risticii (LG085951.6)
Neorickettsia risticii (LF136416.6)
Neorickettsia risticii (HZ486044)
Neorickettsia sennetsu (M73219.1)
Neorickettsia risticii (LG104762.6)
Neorickettsia risticii (LF651990.6)
Neorickettsia risticii (LF134384.6)
Neorickettsia sp. (KX818102)
Neorickettsia sp. (KX818101)
Neorickettsia sp. (KX818100)
Neorickettsia risticii (LV535903.6)
Neorickettsia sennetsu (LG085952.6)
Neorickettsia sennetsu (LF136417.6)
Neorickettsia sennetsu (HZ486045.6)
Neorickettsia risticii (CP001431)
Neorickettsia sennetsu (M73225)
Neorickettsia sp. (LNGI01000001)
Neorickettsia sennetsu (LG104763.6.)
59
Neorickettsia sennetsu (LF651991.6)
Neorickettsia sennetsu (LF134385)
Neorickettsia sennetsu (NR044746)
Neorickettsia sennetsu (NR074386)
Neorickettsia risticii (M21290)
Neorickettsia risticii (AF036653)
Neorickettsia risticii (AF036654)
Neorickettsia risticii (AF037210)
Neorickettsia risticii (AF037211)
Neorickettsia risticii (AF206300)
Neorickettsia risticii (AF380257)
Neorickettsia risticii (AF380258)
Neorickettsia risticii (AY005439)
Neorickettsia risticii (MK760556)
98
Neorickettsia risticii (MK760560)
Neorickettsia risticii str. Illinois (NR074389)
Neorickettsia risticii str. Illinois (NR029162)
Neorickettsia risticii (MK760559)
Neorickettsia sp. (KX818103)
Neorickettsia sp. (KX818106)
63
Neorickettsia sp. (KX462530)
67
Neorickettsia sp. (KF661347)
Neorickettsia risticii (MK760558)
Neorickettsia risticii (MK760557)
65
Neorickettsia risticii (KX001784)
Neorickettsia risticii (AY005441)
Neorickettsia sp. (MK644533)
Neorickettsia sp. (KF878084)
Neorickettsia sp. (KF878083)
Neorickettsia sp. (KF661344)
87
Neorickettsia helminthoeca (KX462531)
72
Neorickettsia helminthoeca (CP007481)
Neorickettsia sp. (KF661350)
50
Neorickettsia sp. (KX818099)
Neorickettsia sp. (KX818105)
Neorickettsia sp. (KF661345)
89
Neorickettsia sp. (KX379497)
Neorickettsia sp. (KX379496)
Neorickettsia sp. (KX818104)
Neorickettsia sp. (KF661346)
62
96
Neorickettsia sp. (KF661349)
Neorickettsia sp. (KF661342)
Neorickettsia sp. (KF661348)
62
Neorickettsia sp. (KF661343)
Neorickettsia sp. (EU780451)
0,0050

Supplementary Fig. S3. Phylogenetic analysis analysis of *Neorickettsia* spp. isolated from horses based on the V2 hypervariable region of the 16S rDNA sequence. GenBank accession numbers have been shown in parentheses. The tree was constructed using the maximum likelihood method, and numbers above the internal nodes indicated the percentages of 1000 bootstrap replicates that supported the branch.

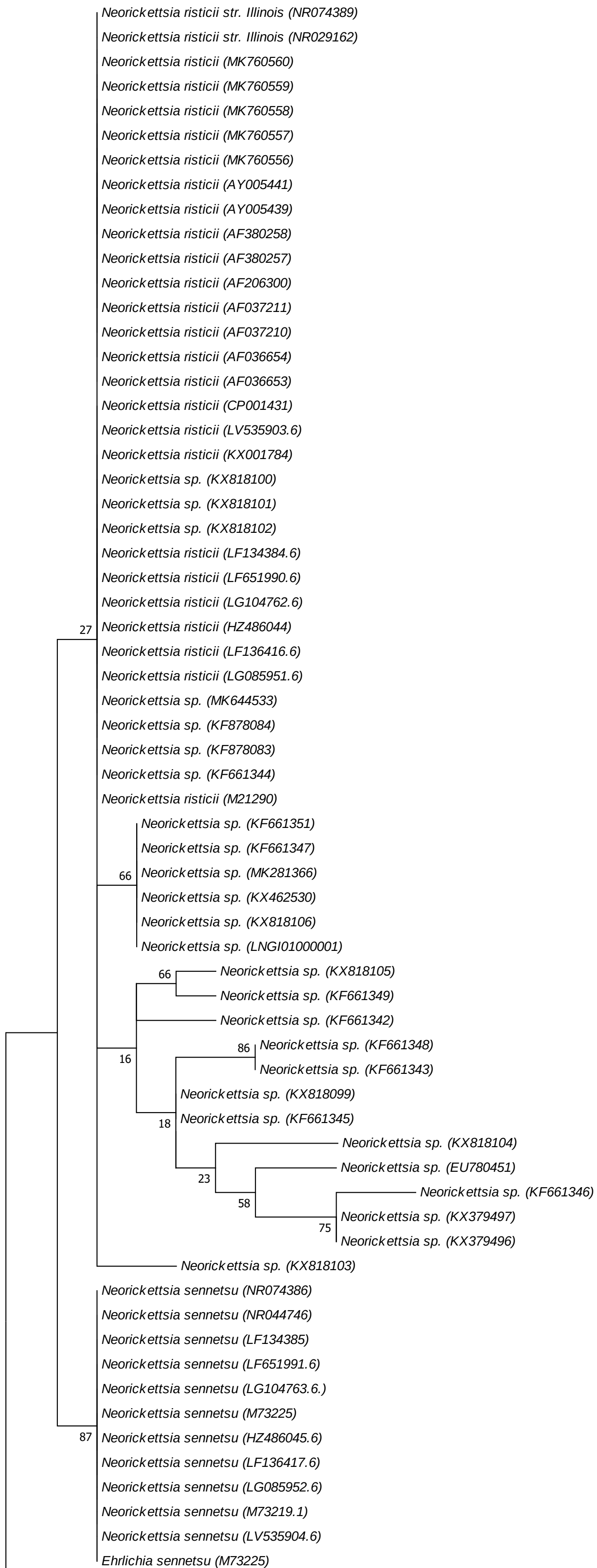
Neorickettsia risticii str. Illinois (NR074389)
Neorickettsia risticii str. Illinois (NR029162)
Neorickettsia risticii (MK760560)
Neorickettsia risticii (MK760559)
Neorickettsia risticii (MK760558)
Neorickettsia risticii (MK760557)
Neorickettsia risticii (MK760556)
Neorickettsia risticii (AY005441)
Neorickettsia risticii (AY005439)
Neorickettsia risticii (AF380258)
Neorickettsia risticii (AF380257)
Neorickettsia risticii (AF206300)
Neorickettsia risticii (AF037211)
Neorickettsia risticii (AF037210)
Neorickettsia risticii (AF036654)
Neorickettsia risticii (AF036653)
Neorickettsia risticii (CP001431)
Neorickettsia risticii (LV535903.6)
Neorickettsia risticii (KX001784)
Neorickettsia sp. (KX818100)
Neorickettsia sp. (KX818101)
Neorickettsia sp. (KX818102)
Neorickettsia risticii (LF134384.6)
Neorickettsia risticii (LF651990.6)
Neorickettsia risticii (LG104762.6)
27
Neorickettsia risticii (HZ486044)
Neorickettsia risticii (LF136416.6)
Neorickettsia risticii (LG085951.6)
Neorickettsia sp. (MK644533)
Neorickettsia sp. (KF878084)
Neorickettsia sp. (KF878083)
Neorickettsia sp. (KF661344)
Neorickettsia risticii (M21290)
Neorickettsia sp. (KF661351)
Neorickettsia sp. (KF661347)
66
Neorickettsia sp. (MK281366)
Neorickettsia sp. (KX462530)
Neorickettsia sp. (KX818106)
Neorickettsia sp. (LNGI01000001)
66
Neorickettsia sp. (KX818105)
Neorickettsia sp. (KF661349)
Neorickettsia sp. (KF661342)
86
Neorickettsia sp. (KF661348)
16
Neorickettsia sp. (KF661343)
Neorickettsia sp. (KX818099)
18
Neorickettsia sp. (KF661345)
Neorickettsia sp. (KX818104)
Neorickettsia sp. (EU780451)
23
Neorickettsia sp. (KF661346)
58
Neorickettsia sp. (KX379497)
75
Neorickettsia sp. (KX379496)
Neorickettsia sp. (KX818103)
Neorickettsia sennetsu (NR074386)
Neorickettsia sennetsu (NR044746)
Neorickettsia sennetsu (LF134385)
Neorickettsia sennetsu (LF651991.6)
Neorickettsia sennetsu (LG104763.6.)
Neorickettsia sennetsu (M73225)
87
Neorickettsia sennetsu (HZ486045.6)
Neorickettsia sennetsu (LF136417.6)
Neorickettsia sennetsu (LG085952.6)
Neorickettsia sennetsu (M73219.1)
Neorickettsia sennetsu (LV535904.6)
Ehrlichia sennetsu (M73225)
Neorickettsia sp. (KF661350)
Neorickettsia helminthoeca (KX462531)
99
Neorickettsia helminthoeca (CP007481)
0,0100

Supplementary Fig. S4. Phylogenetic analysis of *Neorickettsia* spp. isolated from horses based on the V4 hypervariable region of the 16S rDNA sequence. GenBank accession numbers have been shown in parentheses. The tree was constructed using the maximum likelihood method, and numbers above the internal nodes indicated the percentages of 1000 bootstrap replicates that supported the branch.

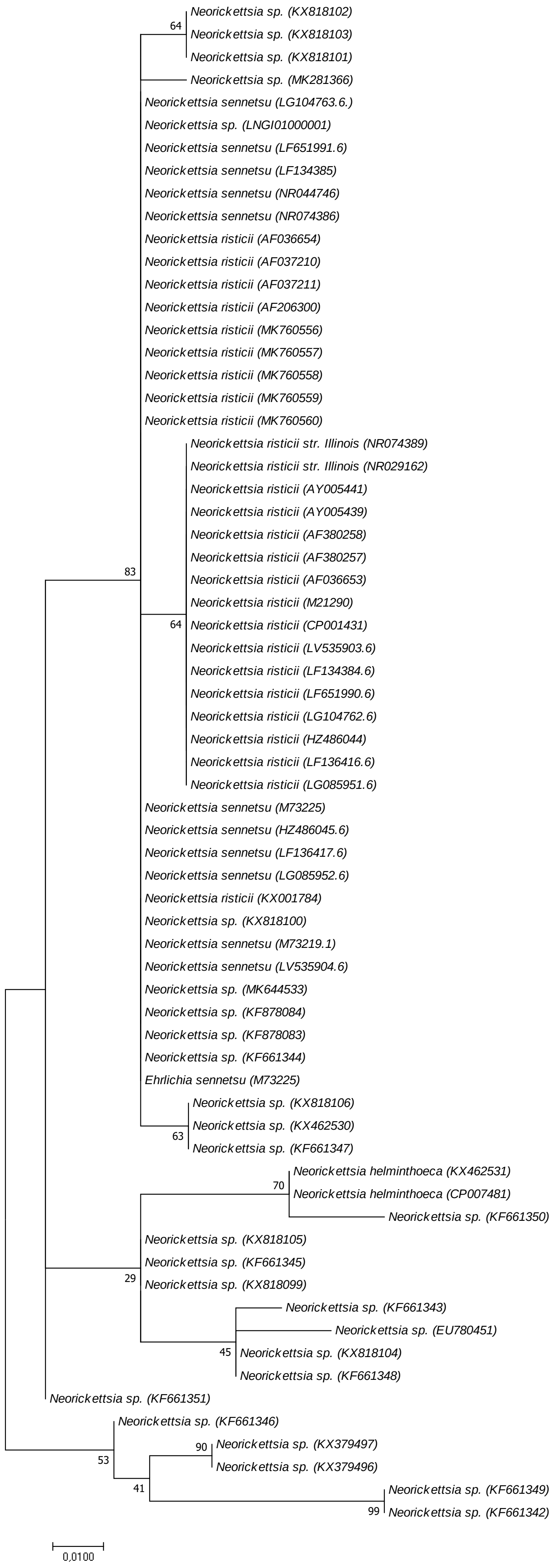

Neorickettsia sp. (KX818102)
Neorickettsia sp. (KX818103)
Neorickettsia sp. (KX818101)
Neorickettsia sp. (MK281366)
Neorickettsia sennetsu (LG104763.6.)
Neorickettsia sp. (LNGI01000001)
Neorickettsia sennetsu (LF651991.6)
Neorickettsia sennetsu (LF134385)
Neorickettsia sennetsu (NR044746)
Neorickettsia sennetsu (NR074386)
Neorickettsia risticii (AF036654)
Neorickettsia risticii (AF037210)
Neorickettsia risticii (AF037211)
Neorickettsia risticii (AF206300)
Neorickettsia risticii (MK760556)
Neorickettsia risticii (MK760557)
Neorickettsia risticii (MK760558)
Neorickettsia risticii (MK760559)
Neorickettsia risticii (MK760560)
Neorickettsia risticii str. Illinois (NR074389)
Neorickettsia risticii str. Illinois (NR029162)
Neorickettsia risticii (AY005441)
Neorickettsia risticii (AY005439)
Neorickettsia risticii (AF380258)
Neorickettsia risticii (AF380257)
Neorickettsia risticii (AF036653)
Neorickettsia risticii (M21290)
Neorickettsia risticii (CP001431)
Neorickettsia risticii (LV535903.6)
Neorickettsia risticii (LF134384.6)
Neorickettsia risticii (LF651990.6)
Neorickettsia risticii (LG104762.6)
Neorickettsia risticii (HZ486044)
Neorickettsia risticii (LF136416.6)
Neorickettsia risticii (LG085951.6)
Neorickettsia sennetsu (M73225)
Neorickettsia sennetsu (HZ486045.6)
Neorickettsia sennetsu (LF136417.6)
Neorickettsia sennetsu (LG085952.6)
Neorickettsia risticii (KX001784)
Neorickettsia sp. (KX818100)
Neorickettsia sennetsu (M73219.1)
Neorickettsia sennetsu (LV535904.6)
Neorickettsia sp. (MK644533)
Neorickettsia sp. (KF878084)
Neorickettsia sp. (KF878083)
Neorickettsia sp. (KF661344)
Ehrlichia sennetsu (M73225)
Neorickettsia sp. (KX818106)
Neorickettsia sp. (KX462530)
Neorickettsia sp. (KF661347)
Neorickettsia helminthoeca (KX462531)
Neorickettsia helminthoeca (CP007481)
Neorickettsia sp. (KF661350)
Neorickettsia sp. (KX818105)
Neorickettsia sp. (KF661345)
Neorickettsia sp. (KX818099)
Neorickettsia sp. (KF661343)
Neorickettsia sp. (EU780451)
Neorickettsia sp. (KX818104)
Neorickettsia sp. (KF661348)
Neorickettsia sp. (KF661351)
Neorickettsia sp. (KF661346)
Neorickettsia sp. (KX379497)
Neorickettsia sp. (KX379496)
Neorickettsia sp. (KF661349)
Neorickettsia sp. (KF661342)
64
83
64
63
70
29
45
90
53
41
99
0,0100

Supplementary Fig. S5. Phylogenetic analysis of *Neorickettsia* spp. isolated from horses based on the V6 hypervariable region of the 16S rDNA sequence. GenBank accession numbers have been shown in parentheses. The tree was constructed using the maximum likelihood method, and numbers above the internal nodes indicated the percentages of 1000 bootstrap replicates that supported the branch.

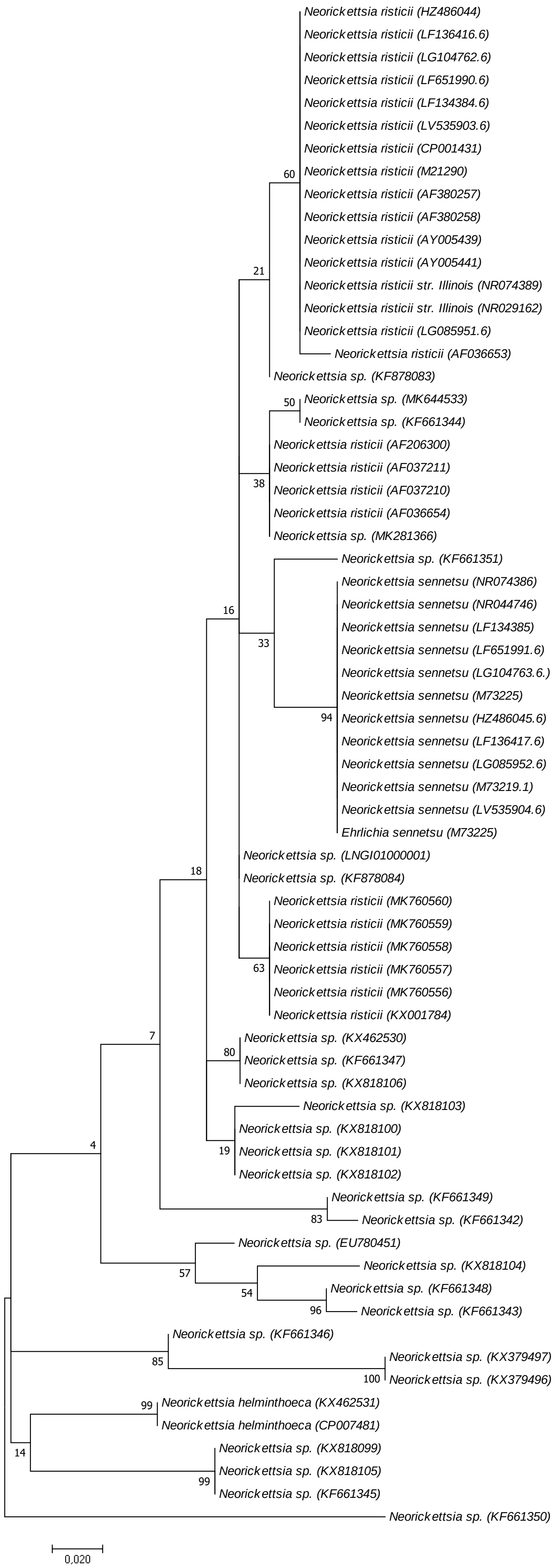
Neorickettsia risticii (HZ486044)
Neorickettsia risticii (LF136416.6)
Neorickettsia risticii (LG104762.6)
Neorickettsia risticii (LF651990.6)
Neorickettsia risticii (LF134384.6)
Neorickettsia risticii (LV535903.6)
Neorickettsia risticii (CP001431)
Neorickettsia risticii (M21290)
Neorickettsia risticii (AF380257)
Neorickettsia risticii (AF380258)
Neorickettsia risticii (AY005439)
Neorickettsia risticii (AY005441)
Neorickettsia risticii str. Illinois (NR074389)
Neorickettsia risticii str. Illinois (NR029162)
Neorickettsia risticii (LG085951.6)
Neorickettsia risticii (AF036653)
Neorickettsia sp. (KF878083)
Neorickettsia sp. (MK644533)
Neorickettsia sp. (KF661344)
Neorickettsia risticii (AF206300)
Neorickettsia risticii (AF037211)
Neorickettsia risticii (AF037210)
Neorickettsia risticii (AF036654)
Neorickettsia sp. (MK281366)
Neorickettsia sp. (KF661351)
Neorickettsia sennetsu (NR074386)
Neorickettsia sennetsu (NR044746)
Neorickettsia sennetsu (LF134385)
Neorickettsia sennetsu (LF651991.6)
Neorickettsia sennetsu (LG104763.6.)
Neorickettsia sennetsu (M73225)
Neorickettsia sennetsu (HZ486045.6)
Neorickettsia sennetsu (LF136417.6)
Neorickettsia sennetsu (LG085952.6)
Neorickettsia sennetsu (M73219.1)
Neorickettsia sennetsu (LV535904.6)
Ehrlichia sennetsu (M73225)
Neorickettsia sp. (LNGI01000001)
Neorickettsia sp. (KF878084)
Neorickettsia risticii (MK760560)
Neorickettsia risticii (MK760559)
Neorickettsia risticii (MK760558)
Neorickettsia risticii (MK760557)
Neorickettsia risticii (MK760556)
Neorickettsia risticii (KX001784)
Neorickettsia sp. (KX462530)
Neorickettsia sp. (KF661347)
Neorickettsia sp. (KX818106)
Neorickettsia sp. (KX818103)
Neorickettsia sp. (KX818100)
Neorickettsia sp. (KX818101)
Neorickettsia sp. (KX818102)
Neorickettsia sp. (KF661349)
Neorickettsia sp. (KF661342)
Neorickettsia sp. (EU780451)
Neorickettsia sp. (KX818104)
Neorickettsia sp. (KF661348)
Neorickettsia sp. (KF661343)
Neorickettsia sp. (KF661346)
Neorickettsia sp. (KX379497)
Neorickettsia sp. (KX379496)
Neorickettsia helminthoeca (KX462531)
Neorickettsia helminthoeca (CP007481)
Neorickettsia sp. (KX818099)
Neorickettsia sp. (KX818105)
Neorickettsia sp. (KF661345)
Neorickettsia sp. (KF661350)
0,020

Supplementary Fig. S6. Phylogenetic analysis of *Neorickettsia* spp. isolated from horses based on the V8 hypervariable region of the 16S rDNA sequence. GenBank accession numbers have been shown in parentheses. The tree was constructed using the maximum likelihood method, and numbers above the internal nodes indicated the percentages of 1000 bootstrap replicates that supported the branch.
